# Integrated analysis of microRNA expression and mRNA transcriptome in lungs of avian influenza virus infected broilers

**DOI:** 10.1186/1471-2164-13-278

**Published:** 2012-06-22

**Authors:** Ying Wang, Vinayak Brahmakshatriya, Blanca Lupiani, Sanjay M Reddy, Benjamin Soibam, Ashley L Benham, Preethi Gunaratne, Hsiao-ching Liu, Nares Trakooljul, Nancy Ing, Ron Okimoto, Huaijun Zhou

**Affiliations:** 1 Department of Animal Science, University of California, Davis, CA, 95616, USA; 2 Department of Veterinary Pathobiology, College of Veterinary Medicine Texas A&M University, Texas, TX, 77840, USA; 3 Department of Biochemistry, University of Houston, Houston, TX, 77004, USA; 4 Department of Animal Science, North Carolina State University, Raleigh, NC, 27695, USA; 5 Department of Animal Science, Texas A&M University, Texas, TX, 77843, USA; 6 Cobb-Vantress, Inc, Siloam Springs, AR, 72761, USA

**Keywords:** Chicken, miRNA, AIV, Deep sequencing, Microarray

## Abstract

**Background:**

Avian influenza virus (AIV) outbreaks are worldwide threats to both poultry and humans. Our previous study suggested microRNAs (miRNAs) play significant roles in the regulation of host response to AIV infection in layer chickens. The objective of this study was to test the hypothesis if genetic background play essential role in the miRNA regulation of AIV infection in chickens and if miRNAs that were differentially expressed in layer with AIV infection would be modulated the same way in broiler chickens. Furthermore, by integrating with parallel mRNA expression profiling, potential molecular mechanisms of host response to AIV infection can be further exploited.

**Results:**

Total RNA isolated from the lungs of non-infected and low pathogenic H5N3 infected broilers at four days post-infection were used for both miRNA deep sequencing and mRNA microarray analyses. A total of 2.6 M and 3.3 M filtered high quality reads were obtained from infected and non-infected chickens by Solexa GA-I Sequencer, respectively. A total of 271 miRNAs in miRBase 16.0 were identified and one potential novel miRNA was discovered. There were 121 miRNAs differentially expressed at the 5% false discovery rate by Fisher’s exact test. More miRNAs were highly expressed in infected lungs (108) than in non-infected lungs (13), which was opposite to the findings in layer chickens. This result suggested that a different regulatory mechanism of host response to AIV infection mediated by miRNAs might exist in broiler chickens. Analysis using the chicken 44 K Agilent microarray indicated that 508 mRNAs (347 down-regulated) were differentially expressed following AIV infection.

**Conclusions:**

A comprehensive analysis combining both miRNA and targeted mRNA gene expression suggests that gga-miR-34a, 122–1, 122–2, 146a, 155, 206, 1719, 1594, 1599 and 451, and MX1, IL-8, IRF-7, TNFRS19 are strong candidate miRNAs or genes involved in regulating the host response to AIV infection in the lungs of broiler chickens. Further miRNA or gene specific knock-down assay is warranted to elucidate underlying mechanism of AIV infection regulation in the chicken.

## Background

Avian influenza virus (AIV) infection is a world-wide threat to both human and avian species. AIV causes an infection of the respiratory tract of the host, triggering a cascade of innate and adaptive immune responses. Great efforts have been made to develop new intervention strategies to control AIV infections in chickens [1,2]. However, more effective measures against AIV infection in the chicken are still needed. AI outbreaks in chickens could not only cause dramatic economic losses to the poultry industry, but also threaten human health. Therefore, understanding host response to AIV infection and chicken-virus interaction is not only essential to the poultry industry, but also provides key insights into the prophylactic and therapeutic protection for other influenza hosts including humans.

miRNAs are short, 17–24 nt RNAs, which comprise a large family of regulatory molecules found in almost all multi-cellular organisms [3]. These small RNAs have been demonstrated to have important functions in a variety of biological processes and have been implicated in many diseases including influenza, hepatitis and cancer [4-10]. miRNAs are capable of regulating mammalian immune cell differentiation, the outcome of immune responses to infection, and the development of diseases of immunological origins [11]. There are multiple mechanisms of miRNA-mediated regulation of gene expression including translational repression, disruption of mRNA stability, miRNA-mediated deadenylation and inhibition of polypeptide elongation [12]. Determining how and when miRNA suppress target mRNA gene expression remains one of the greatest challenges in the field.

Through recognition of sequence-complementary target elements, miRNAs can either translationally suppress or catalytically degrade both cellular and viral RNAs [3,13]. Host miRNAs are able to impinge on viral life cycles, viral tropism, and the pathogenesis of viral diseases [14]. miRNAs can potentially regulate different steps of a virus life cycle and abrogate toxicities of replication-competent viruses [15-18]. For example, human miR-32 represses the replication of the retrovirus primate foamy virus type 1 (PFV-1) through the down-regulation of replication-essential viral proteins encoded by open reading frame 2 (ORF2) [16]. Based on computational prediction, human miR-136 and miR-507 have potential binding sites at the polymerase basic 2 (PB2) and hemmagglutinin (HA) proteins of H5N1 AIV, and those two miRNAs may modulate AIV infection in humans [19].

Next generation sequencing (NGS, deep sequencing) has provided a powerful tool to identify differentially expressed miRNAs especially low abundance ones under conditions of physiological perturbation. We previously used a Solexa Sequencer to identify differentially expressed chicken miRNAs in AIV infected lungs and trachea of layer type birds [20]. Genetics play a significant role in host response to viral infection. We hypothesize that gene expression of host cellular miRNAs following virus infection could be different between different chicken genetic lines. There are two major types of chickens: broilers (meat type chickens) and layers (egg type chickens). In the current study, a deep sequencing approach was employed to identify differentially expressed miRNAs with AIV infection in broilers.

Identification of differentially expressed host miRNAs is just the first step towards understanding miRNA regulation of host-virus interactions, and then underlying mechanisms that how genes targeted by differentially expressed miRNAs mediate host-virus interaction would be desired. Dissection of miRNA modulation of both host and viral mRNA expression will provide insights in the cellular mechanisms of host-virus interaction. A powerful symbiosis between microarrays and NGS technologies has been witnessed [21]. Therefore, global gene expression (mRNA) profiling of host response to AIV infection was conducted to identify potential genes associated with AIV infection using a chicken 44 K Agilent microarray. We integrated predicted target genes information (based on differentially expressed miRNAs) with differentially regulated mRNA affected by AIV infection to understand how miRNAs regulate mRNA gene expression with AIV infection in the chicken.

## Results

### Virus titers in lungs

Virus replication in lungs was examined using real-time RT-PCR by measuring influenza virus matrix gene from total RNAs at 4 days post inoculation (dpi). Virus titers in the four infected chicken, determined by extrapolation of real-time RT-PCR data, were 1.69, 3.41, 3.81, and 4.52 log_10_ EID_50_/ml. Lung samples from all 4 non-infected chickens were negative.

### miRNA sequences from small RNA libraries

A total of 2,672,582 and 3,318,307 filtered high quality reads were obtained from infected and non-infected chicken, respectively (Table 1). In the library from infected chicken lungs, 2,314,793 of these reads were exact matches and another 357,789 reads were loose matches to known chicken miRNAs. In the library of non-infected chicken lungs, 2,875,366 of these reads were exact matches and another 442,941 reads were loose matches to known chicken miRNAs. All reads with a perfect match to mature miRNA sequences from chicken deposited in miRBase version 16.0 ( http://microrna.sanger.ac.uk/) [22-24] with insertions or deletions of 1–4 nucleotides at the 5’ and 3’ ends of miRNAs were considered to represent Dicer-processing products from each of the chicken miRNA precursors [20]. The loose match reads were defined as no more than 4 nt differences comparing to the known chicken miRNA sequences determined as we did previously [20]. The sum of exact and loose match reads was used as the total number of reads for each miRNA.

**Table 1:** Number of reads of microRNAs from lungs of AIV infected and non-infected chickens

	**Infected lung**	**Non-infected lung**
High quality/both adapter	2,672,582	3,318,307
Exact match to known chicken miRNAs	2,314,793	2,875,366
Loose match to known chicken miRNAs	357,789	442,941

**Figure 1:**
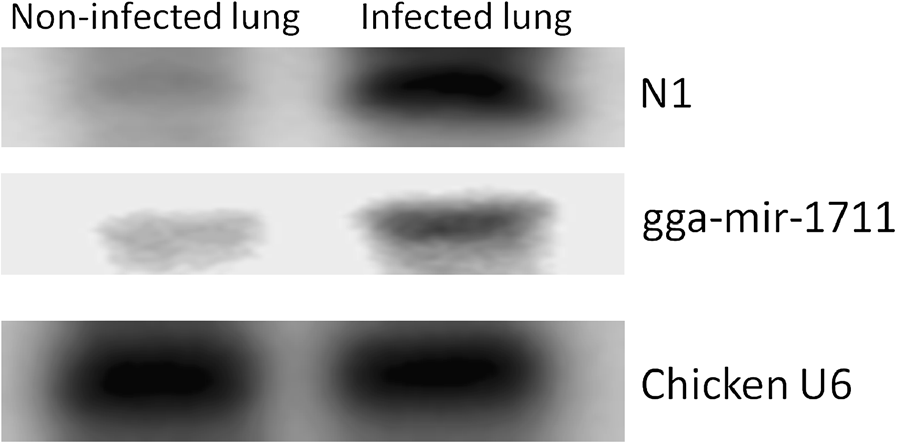
**Confirmation of miRNAs.** Northern blot analysis was performed to confirm the presence of a novel miRNA (N1) and another known chicken miRNA (gga-miR-1711) in infected and non-infected chicken lungs. U6 probe was used as a control.

Of the 499 distinct *Gallus gallus* (gga) miRNA entries in miRBase [22-24], 272 miRNAs were identified in the current chicken lung small RNA library. Also, one potential novel miRNA was identified (Additional file 1: Table S1). Differential expression of this novel miRNA “N1” and chicken miRNA miR-1711 were confirmed by Northern blot analysis (Figure 1).

### miRNA expression profiling analysis

**Figure 2:**
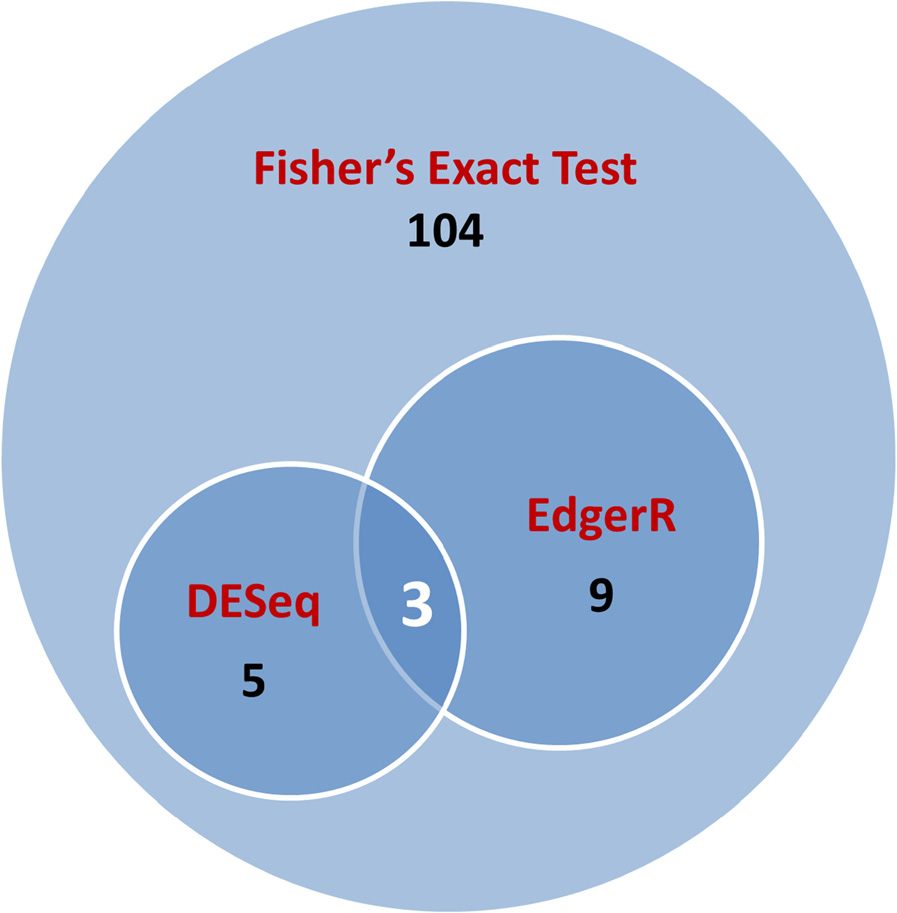
**Integration of numbers of differentially expressed miRNAs by different statistical methods.** Differentially expressed miRNAs were identified by Fisher exact test, DESeq and edgeR, respectively (P < 0.05, fold change > 2).

miRNA expression profiles of infected and non-infected chicken lungs were compared. Differentially expressed miRNAs were identified (P < 0.05, Q < 0.05 and fold change > 2) by Fisher’s exact test. Between infected and non-infected lungs, 121 miRNAs were differentially expressed. Of those, 43 miRNAs were unique to infected lung and 8 miRNAs were unique to uninfected lung. Sixty-five miRNAs were more highly expressed in infected lungs, while 5 miRNAs were more highly expressed in non-infected lungs (Table 2). With limited biological replicates for the nature of next generation sequencing technology, there is no standard method available for this type of analysis. Two newly developed methods (more conservative): DESeq and edgeR, were implemented in this study. The differentially expressed miRNAs are presented in Tables 3 and 4, there were 8 and 12 miRNAs differentially expressed (P < 0.05, fold change > 2) by DESeq and edgeR, respectively, and all these miRNAs were identified by the Fisher’s exact test (Figure 2). Three out of eight miRNAs by DESeq analysis were also identified by edgeR. All miRNAs identified by DESeq were up-regulated with AIV infection. For the 12 miRNAs identified by the edgeR program, nine miRNAs specifically expressed in the infected chickens and one miRNA in non-infected birds; two miRNAs were up-regulated with AIV infection.

**Table 2:** Differentially expressed miRNAs between lungs of infected and non-infected chickens (P < 0.05, Q < 0.05 and Ratio > 2) by Fisher’s exact test.

**miRNA**	**Position on chromosomes**	**Reads in infected**	**Reads in non-infected**	**Ratio infected/non-infected (Normalized)**
gga-miR-1719	chr12:842924-843012	109	0	−^1^
gga-miR-1585	chr19:8800028-8800118	65	0	-
gga-miR-1777	chr28:2555498-2555592	24	0	-
gga-miR-460b-5p	chr4:26873962687485	23	0	-
gga-miR-1716	chr5:60283968-60284072	22	0	-
gga-miR-3537	chr6:18024717-18024793	19	0	-
gga-miR-1718	chr5:33777662-33777741	18	0	-
gga-miR-1354	chr4: 3970359-3970439	18	0	-
gga-miR-1792	chr3:7712006-7712104	16	0	-
gga-miR-3535	chr9:16372628-16372709	15	0	-
gga-miR-1610	chr8:12398260-12398347	15	0	-
gga-miR-1631	chrZ:15789429-15789502	13	0	-
gga-miR-1805-5p	chr1:135141607-135141690	12	0	-
gga-miR-1604	chr1:312691-312787	12	0	-
gga-miR-153	chr2:8765687-8765773	12	0	-
gga-miR-1593	chr1:61691788-61691877	12	0	-
gga-miR-3538-1	chrUn:44040736-44040810	10	0	-
gga-miR-3538-2	chr1:52608155-52608229	10	0	-
gga-miR-1723	chr2:41377973-41378078	10	0	-
gga-miR-1584	chrZ:18238506-18238570	10	0	-
gga-miR-1754	chr9:25014275-25014342	9	0	-
gga-miR-3528	chr17:8404342-8404438	9	0	-
gga-miR-1644	chr14:8284308-8284393	8	0	-
gga-miR-1745-1	chr24:5271413-5271449	8	0	-
gga-miR-1770	chr2:151547087-151547183	7	0	-
gga-miR-1809	chr8:23417849-23417956	7	0	-
gga-miR-34a	chr21:3251514-3251622	7	0	-
gga-miR-1681	chr2:96361604-96361703	6	0	-
gga-miR-1692	chr9:23692587-23692675	6	0	-
gga-miR-1805-3p	chr1:135141607-135141690	6	0	-
gga-miR-1463	chr5:11171642-1171751	6	0	-
gga-miR-1560	chr11:20587431-20587444	6	0	-
gga-miR-1700	chr1:140966218-140966317	6	0	-
gga-miR-1712	chr3:81937337-81937409	6	0	-
gga-miR-1772	chr6:11560478-11560546	6	0	-
gga-miR-1713	chr7:17384289-17384387	6	0	-
gga-miR-1781	chr14:3330762-3330854	6	0	-
gga-miR-1551	chr14:5233361-5233450	5	0	-
gga-miR-2127	chr1:170154815-170154918	5	0	-
gga-miR-3527	chrMT:8673-8781	5	0	-
gga-miR-3533	chrUn:20438961-20439044	5	0	-
gga-miR-3536	chr25:1478485-1478562	5	0	-
gga-miR-1785	chr11:20641236-20641337	5	0	-
gga-miR-1594	chrZ:75709-75799	473	17	34.54
gga-miR-1599	chr7: 25926968-25927029	184	7	32.64
gga-miR-1767	chr3:44732913-44732971	71	7	12.59
gga-miR-1662	chr2:1721334-1721406	140	17	10.23
gga-miR-202	chr6:22813068-22813156	40	5	9.93
gga-miR-122-1	chrZ: 649337-649413	1952	279	8.69
gga-miR-1766-1	chr2:77319215-77319307	42	6	8.69
gga-miR-122-2	chrUn:12066796-12066872	1685	258	8.11
gga-miR-32	chr2:86506451-86506520	45	7	7.98
gga-miR-204-2	chr10:6651274-6651374	12	2	7.45
gga-miR-211	chr28:1784394-1784467	12	2	7.45
gga-miR-451	chr19: 5823968-5824036	207487	35518	7.25
gga-miR-19b	chr1: 152248183-152248269	955	181	6.55
gga-miR-1694	chr7:5419755-5419852	26	5	6.46
gga-miR-1729	chr15:769596-769666	4292	843	6.32
gga-miR-1611	chr10:16350472-16350560	167	38	5.46
gga-miR-2188	chr22:2684926-2685094	7045	1800	4.86
gga-miR-18a	chr1:152248626-152248718	88	23	4.75
gga-miR-1581	chr1:51158137-51158222	18	5	4.67
gga-miR-193b	chr14: 759453-759535	159	47	4.20
gga-miR-1451	chr3:78710207-78710207	129	42	3.81
gga-miR-1587	chr19:1782806-1782901	15	5	3.72
gga-miR-1572	chr12:9668820-9668820	182	61	3.70
gga-miR-3523	chr13:8968882-8969047	65	22	3.67
gga-miR-18b	chr4:3970228-3970311	70	24	3.62
gga-miR-155	chr1:105930213-105930275	40	14	3.55
gga-miR-454	chr15:399833-399953	31	11	3.50
gga-miR-15a	chr1: 173700493-173700575	2413	861	3.48
gga-miR-144	chr19: 5824123-5824207	13216	4727	3.47
gga-miR-551	chr9:21966405-21966517	25	9	3.45
gga-miR-218-1	chr4:77774698-77774806	11	4	3.41
gga-miR-218-2	chr13:4322806-4322954	11	4	3.41
gga-miR-193a	chr18: 6423770-6423846	1230	461	3.31
gga-miR-223	chr4: 232949-233048	1842	717	3.19
gga-miR-30b	chr2:148331598-148331684	165	67	3.06
gga-miR-214	chr8:4739550-4739659	74	32	2.87
gga-miR-142-3p	chr19: 496983-497070	1055	461	2.84
gga-miR-142-5p	chr19: 496983-497070	1100	481	2.84
gga-miR-106	chr4: 3970359-3970439	897	394	2.83
gga-miR-16-2	chr9:23742791-23742884	1952	856	2.83
gga-miR-16-1	chr1: 173700351-173700434	2724	1206	2.80
gga-miR-1579	chr6:3677284-3677350	164	73	2.79
gga-miR-20a	chr1: 152248306-152248403	426	190	2.78
gga-miR-1416	chrZ: 34596479-34596567	25	12	2.59
gga-miR-146a	chr13: 7555593- 7555691	1331	639	2.59
gga-miR-1798	chr20:9654914-9655009	36	18	2.48
gga-miR-3531	chr23:417154-417240	22	11	2.48
gga-miR-20b	chr4:3970047-3970131	734	378	2.41
gga-miR-1434	chr28:1055204-1055280	89	46	2.40
gga-miR-29a	chr1: 3236329-3236417	205	108	2.36
gga-miR-29c	chr26: 2511658-2511746	205	108	2.36
gga-miR-24	chrZ:41158175-41158242	10052	5343	2.34
gga-miR-7b	chrUn:38163821-38163930	251	134	2.33
gga-miR-17-5p	chr1:152248781-152248865	1831	982	2.32
gga-miR-15c	chr4:4049055-4049130	1002	538	2.31
gga-miR-1763	chr14:12895655-12895720	147	80	2.28
gga-miR-23b	chrZ:41157406-41157491	15783	8718	2.25
gga-miR-147-1	chr1:12334922-12334991	92	52	2.20
gga-miR-17-3p	chr1:152248781-152248865	1480	853	2.15
gga-miR-1800	chr5:47604931-47605006	98	58	2.10
gga-miR-458	chr13:8034158-8034273	69	41	2.09
gga-miR-92	chr1:152248070-152248417	13819	8291	2.07
gga-miR-7-1	chrZ:39554766-39554874	81	49	2.05
gga-miR-1705	chr17:9510405-9510494	26	16	2.02
gga-miR-7-2	chr10:14823525-14823623	71	44	2.00
gga-miR-1306	chr15:1296916-1296984	20	62	0.40
gga-miR-206	chr3: 110390439-110390514	28	98	0.35
gga-miR-301	chr15:406313-406405	5	19	0.33
gga-miR-1638	chr5:58712377-58712463	5	23	0.27
gga-miR-187	chr2:85892470-85892555	6	33	0.23
gga-miR-449b	chrZ: 16040763-16040856	0	23	0^2^
gga-miR-460a	chr2:3583690-3583779	0	11	0
gga-miR-1765	chr18:5840573-5840677	0	9	0
gga-miR-216c	chr3:288216-288301	0	7	0
gga-miR-1607	chr2: 45452355-45452433	0	6	0
gga-miR-1555	chr1:149148336-149148421	0	6	0
gga-miR-1c	chr7:36625855-36625928	0	6	0
gga-miR-3529	chr10:14823529-14823619	0	6	0

Note: ^1^ Specifically expressed in infected lungs.

^2^ Specifically expressed in non-infected lungs.

**Table 3:** Differentially expressed miRNAs between lungs of infected and non-infected chickens (P < 0.05 and Ratio > 2) by DESeq

	**DESeq**		**Fisher’s exact test**	
**miRNA**	**P-value**	**Fold(I/C)**	**P-value**	**Fold(I/C)**
gga-mir-1719	0.01	-	0.00	−^1^
gga-mir-1594	0.00	24.70	0.00	34.55
gga-mir-1599	0.02	23.33	0.00	32.64
gga-mir-122-1	0.01	6.21	0.00	8.69
gga-mir-122-2	0.02	5.80	0.00	8.11
gga-mir-451	0.01	5.19	0.00	7.25
gga-mir-19b	0.04	4.68	0.00	6.55
gga-mir-1729	0.03	4.52	0.00	6.32

**Table 4:** Differentially expressed miRNAs between lungs of infected and non-infected chickens (P < 0.05 and Ratio > 2) by edgeR

	**edgeR**		**Fisher’s exact test**	
**miRNA**	**P-value**	**Fold(I/C)**	**P-value**	**Fold(I/C)**
gga-mir-1719	0	433.20	0	−^1^
gga-mir-1585	0.01	258.74	0	-
gga-mir-1777	0.03	96.16	0	-
gga-mir-460b	0.03	92.20	0	-
gga-mir-1716	0.03	88.23	0	-
gga-mir-3537	0.04	76.34	0	-
gga-mir-1354	0.04	72.37	0	-
gga-mir-1718	0.04	72.37	0	-
gga-mir-1792	0.05	64.44	0	-
gga-mir-1594	0.02	26.96	0	34.54
gga-mir-1599	0.02	24.98	0	32.64
gga-mir-449b	0.03	−100	0	0^2^

Note: ^1^ Specifically expressed in infected lungs.

^2^ Specifically expressed in non-infected lungs.

**Figure 3:**
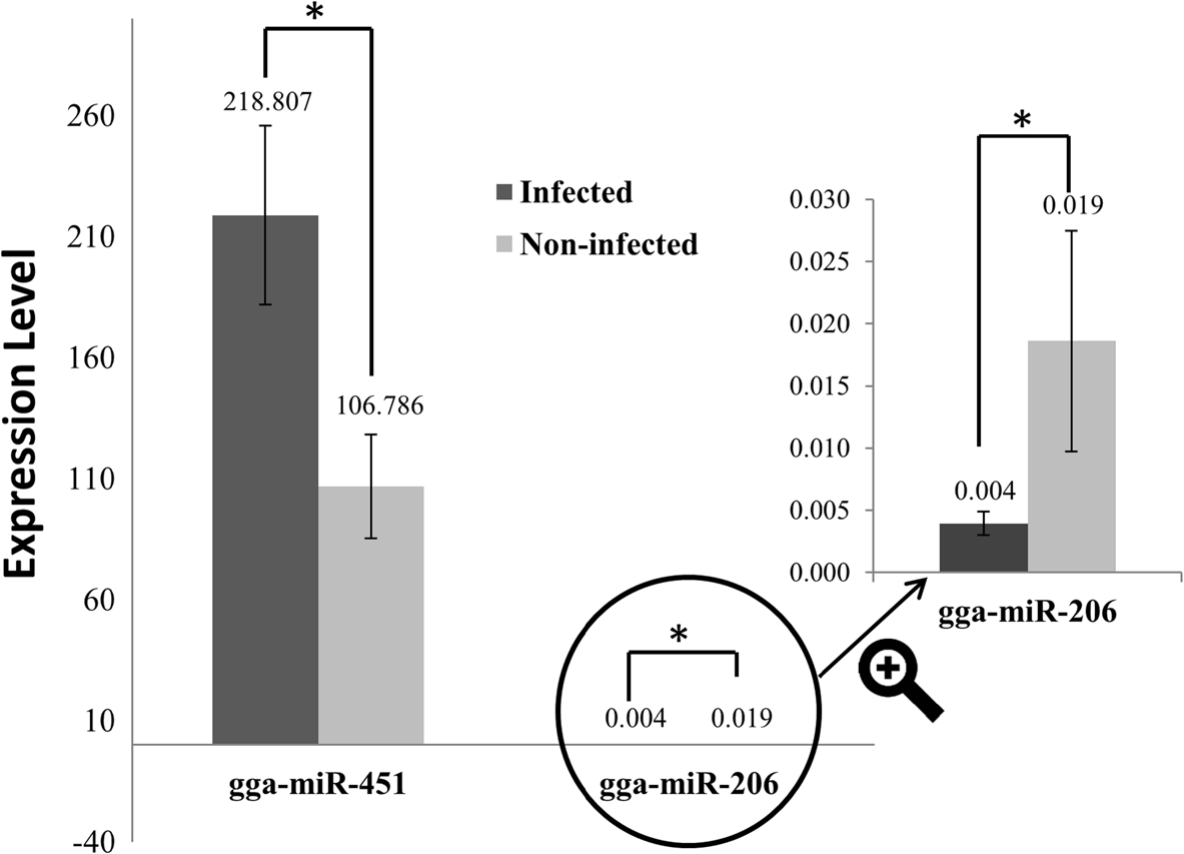
**Validation of two differentially expressed miRNAs by TaqMan miRNA assays.** Two differentially expressed miRNAs (gga-miR-206 and 451) identified by deep sequencing were confirmed by using TaqMan miRNA assays.* *P* <0.05.

TaqMan miRNA assays were used to confirm two differentially expressed miRNAs identified by deep sequencing. There was general consistency between the TaqMan assays and deep sequence analysis of miR-451 and miR-206 in terms of direction of regulation and statistical significance (Figure 3). Specifically, there was a 2.05 fold up-regulation (7.25 fold in deep sequencing analysis) in miR-451, and 4.71 fold down regulation (2.86 fold in deep sequencing analysis) in miR-206 with AIV infection in lungs (P < 0.05).

### miRNA target identification and validation

Potential targets of differentially expressed miRNAs were predicted by the miRNA target prediction algorithm miRanda 3.1 [25]. The entire list of immune target genes related to AIV infection is listed in Additional file 2: Table S2. One hundred and seventy-one immune related genes were predicted to be targets of 35 differentially expressed miRNAs. Each miRNA can target hundreds of genes. We are especially interested in the targeted immune-related genes; some immune related genes each had several predicted miRNA binding sites. For example, IL-17 receptor D (Accession No.: AY278204), has predicted binding sites for seven differentially expressed miRNAs: gga-miR-30b, 34a, 142-5p, 202, 460b-5p, 449b, and 460a. Interestingly, first five miRNAs were up-regulated, while last two miRNAs were down-regulated following AIV infection. This might explain why IL-17 receptor D was not differentially expressed if these differentially expressed miRNA regulate in different direction.

**Figure 4:**
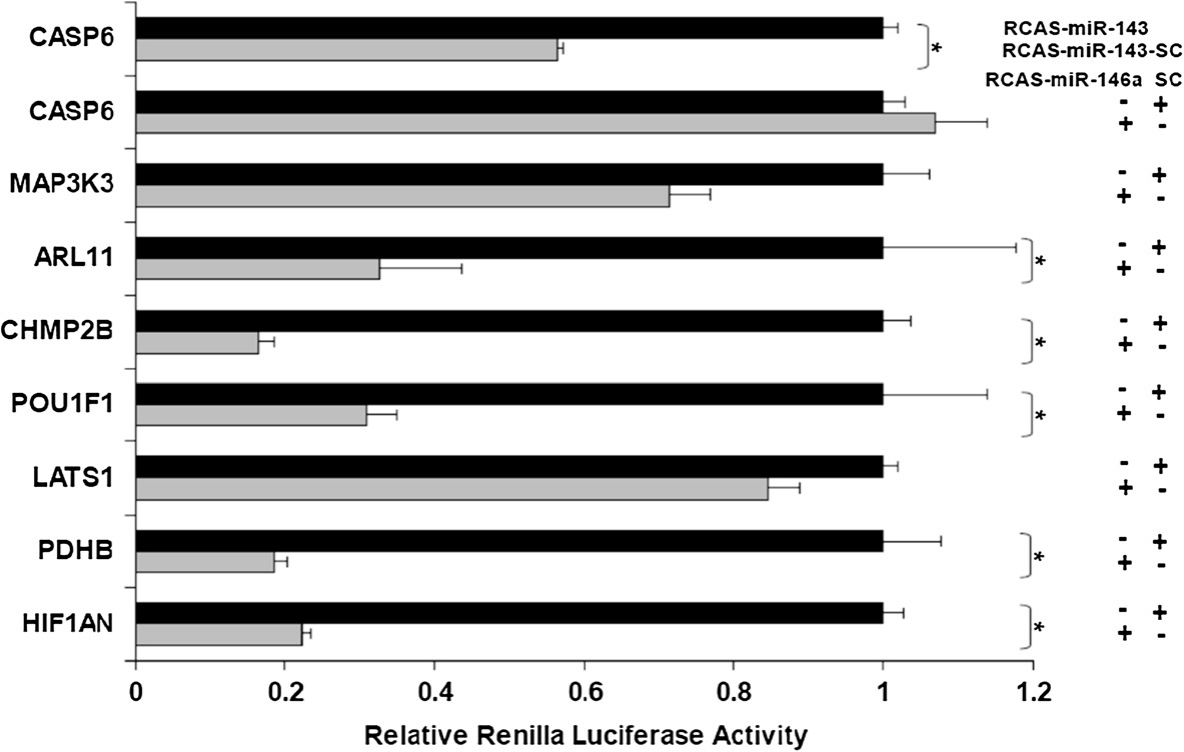
**Validation of miR-146a target genes in the Renilla luciferase reporter system.** Seven potential *miR-146a* target genes predicted by the miRanda algorithm were chosen for validation. For each predicted target gene a luciferase reporter vector was constructed in which the predicted *miR-146a* binding site was cloned into the 3’ UTR of a *Renilla* luciferase reporter gene. The *Renilla* luciferase activities were normalized to *Firefly* luciferase activities (under the control of an independent promoter). The relative expression of each *Renilla* luciferase target construct was compared between cells expressing *miR-146a* and those expressing the scrambled control sequence (SC) using a *t-test* for statistical significance (p < 0.05). Error bars indicate standard deviation. CASP6, a gene containing no binding site for *miR-146a* but predicted to contain a *miR-143* target site was used as negative control.

Of particular note, gga-miR-146a is one of differentially expressed miRNAs that was associated with virus infection in both broiler (current study) and layer chickens [20]. Seven potential target genes (Table 5) of gga-miR-146a were picked for the validation by a dual luciferase reporter assay. The results are shown in Figure 4. The 3’ UTR of five (ARL11, CHMP2B, POU1F1, PDHB and HIF1AN) out of the seven genes targeted by miR-146a showed significant suppression of *Renilla* luciferase activity in RCAS-miR-146a infected cells relative to those infected with RCAS-SC (P < 0.05). Inhibition of the luciferase activity of significant targets varied between 65–85% amongst target sites.

**Table 5:** Potential gga-miR-146a targets

**Symbol/GI**	**gga-miR-146a:mRNA(3’UTR) interaction**	**miRanda score/energy (kcal/mol)**	**Binding site**	Insert location*
HIF1AN/118092762	3' UUGGGUACCUUAAGUCAAGAGU 5'|:|:: ||| || ||||||||5'AGCTTCTGG--TTGAGTTCTCA 3'	167/-18.0	2261-2280	1959-2417
PDHB/118097022	3' UUGGG--UACCUUAAGUCAAGAGU 5'|:||: | || | ||||||||||5'AGCCTAAAAGGCA-TCAGTTCTCA 3'	168/-22.3	3732-3754	3365-3864
LATS1/118088356	3' UUG--GGUACCUUAA----GUCAAGAGU 5'|:| |::||||| | :||||||||5' AGCTGCTGTGGAAATGGCATAGTTCTCA 3'	167/-20.5	4243- 4270	4067-4381
POU1F1/45383513	3' UUGGGUACCUUAAGUCAAGAGU 5'| :|: | |: | ||||||||5'ACTCTCTTCAGGT-AGTTCTCA 3'	150/-16.5	2979-2999	2327-3057
CHMP2B/71896762	3' UUGGGUAC-CUUAAGUCAAGAGU 5'|| :| || |||| |||||||||5'AAGTC-TGAGAATGCAGTTCTCA 3'	174/-21.1	1893-1914	1778-2027
ARL11/118084874	3' UUGG-GUA---CCUUAAGUCAAGAGU 5'||| ||| ||| ||||||||5'GACCGCATATAGGA----AGTTCTCA 3'	157/-19.1	1579-1600	1424-1694
MAP3K3/118102843	3' UUGGGU----A-CCUUAAG----UCA-AGAGU 5'::|||| | ||||| : ||| |||||5'GGCCCAAGAGTGGGAATGTAAGAAGTGTCTCA 3'	127/-19.6	2235-2266	2057-2320

Note: * The 3’UTR predicted target genes containing gga-miR-146a binding sites were cloned into the 3’UTR of the psiCHECK-2 vector (Promega).

Recent evidence indicates that cellular miRNAs can also target viral genes [16]. Potential viral genes targeted by differentially expressed miRNAs were predicted using Vita program [26]. All of the AIV genes were predicted to be targeted by at least one of up or down regulated miRNAs (Table 6). For example, the gga-miR-34a, which was only expressed in infected chicken lungs, not only had 14 immune related target genes (Additional file 2: Table S2), but also targeted the AIV HA, NA, PA, PB1 and PB2 genes. In general, more AIV genes were targeted by induced host miRNAs than repressed miRNAs (8.33 times higher). Some miRNAs had only two viral targets, such as gga-mir-32 (targeting HA and NS genes) and gga-miR-30b (targeting M and NA genes). Some differentially expressed miRNAs had multiple predicted viral targets, such as gga-miR-202 which is predicted to target all nine AIV genes.

**Table 6:** AIV viral targets of differentially expressed miRNAs between lungs of infected and non-infected chicken (P < 0.05, Q < 0.05 and Ratio > 2)

**miRNA**	**Ratio of Infected/Non-infected (Normalized)**	**AIV RNA segments**
gga-miR-153	Specifically expressed in infected lung	HA, NA, PA, PB1 and PB2
gga-miR-34a	Specifically expressed in infected lung	HA, NA, PA, PB1 and PB2
gga-miR-202	+9.93	HA, M, NA, NP, NS, PA, PB1 and PB2
gga-miR-32	+7.98	HA and NS
gga-miR-211	+7.45	HA, M, NA, NP, NS, PA, PB1 and PB2
gga-miR-19b	+6.55	HA, NS, PA and PB1
gga-miR-18a	+4.75	HA, M, NA, PB1 and PB2
gga-miR-18b	+3.62	HA, M, NA, PB1 and PB2
gga-miR-155	+3.55	HA, NA, NP, NS and PB1
gga-miR-15a	+3.48	HA, M NP, NS and PB2
gga-miR-223	+3.19	HA, NA, PB1 and PB2
gga-miR-30b	+3.06	M and NA
gga-miR-142-3p	+2.84	HA, NA, PA, PB1 and PB2
gga-miR-106	+2.83	HA, NA, PA, PB1 and PB2
gga-miR-20a	+2.78	HA, NA, NP, PB1 and PB2
gga-miR-146a	+2.59	HA, M, NA, NP, NS, PA, PB1 and PB2
gga-miR-20b	+2.41	HA, M, NA, NP, NS, PB1 and PB2
gga-miR-29a	+2.36	HA, M, NA, NP, PA, PB1 and PB2
gga-miR-29c	+2.36	HA, M, NA, NP, PA and PB1
gga-miR-24	+2.34	HA, M, NA, NP, NS, PA, PB1 and PB2
gga-miR-7b	+2.33	HA, M, NA, PA, PB1
gga-miR-17-5p	+2.32	HA, M, NA, NP, PA, PB1 and PB2
gga-miR-23b	+2.25	HA, M, PA and PB1
gga-miR-17-3p	+2.15	HA, M, NA, NP, PA and PB2
gga-miR-92	+2.07	HA, M, NP, NS, PB2
gga-miR-206	−2.86	HA, NA, NP, PB1 and PB2
gga-miR-301	−3.03	HA, NA, PB1 and PB2
gga-miR-187	−4.35	NA, NP, PB1 and PB2

Note: + Up-regulated with AIV infection; - Down-regulated with AIV infection.

### Host mRNA profile analysis

The genome-wide expression profiling of host response to AIV infection was carried out using chicken 44 K Agilent microarray. There were 508 genes differentially expressed (161 up-regulated vs. 347 down-regulated) between AIV infected vs. non-infected chickens (P < 0.05, Fold-change > 1.5). The fold-change of gene expression between infected and non-infected group ranged from 34.33 to −10.10.

The integrative differentially expressed miRNAs and mRNA expression of its potential targeted immune-related genes are presented in Table 7. Eight immune related host genes were significantly up or down regulated with AIV infection. Four genes were significantly up-regulated, while the rest were significantly down-regulated. Chicken MX1 gene, which was reported to be associated with influenza virus resistance [27], had the highest fold-change (11.46 fold) followed by interleukin 8 [28] (11.03 fold) and interferon regulatory factory 7 [29] (2.11 fold). Tumor necrosis factor receptor superfamily member 19 was down-regulated [30] (1.85 fold). In general, positive correlations between miRNAs and mRNA expression were observed.

**Table 7:** Differentially expressed immune related host mRNAs between lungs of infected and non-infected chickens (P < 0.05 and Fold-change > 1.5)

**Gene description**	**Gene Accession**	**Infected vs. Non-infected (Fold-change)**	**miRNA**^**1**^**(fold change)**
MX1 myxovirus (influenza virus) resistance 1 [27]	Z23168	+11.46	gga-miR-155(+3.55)
			gga-miR-206(−2.86)
Interleukin 8 (IL8) [28]	M16199	+11.03	gga-miR-32(+7.98)
Interferon regulatory factor 7 (IRF7) [29]	U20338	+2.11	gga-miR-142-5p(+2.84)
Interleukin1receptor-like1, transcript variant1 [51]	AB041738	+1.65	gga-miR-460 (only expressed in infected lungs)
TNF receptor superfamily, member 19 [30]	BX931334	−1.85	gga-miR-187(−4.35)
Tipartite motif-containing 7.1 [52]	BX934475	−1.81	NA^2^
RAC serine/threonine-protein kinase3 (ATK3) [53]	BX950472	−1.65	NA
C-fringe 1 [54]	U97157	−1.52	NA

Note: ^1^miRNAs targeting on differentially expressed immune related genes; ^2^ No miRNAs targeting on the gene; +: Up-regulated with AIV infection; –: Down- regulated with AIV infection.

### Gene ontology (GO) analysis

**Figure 5:**
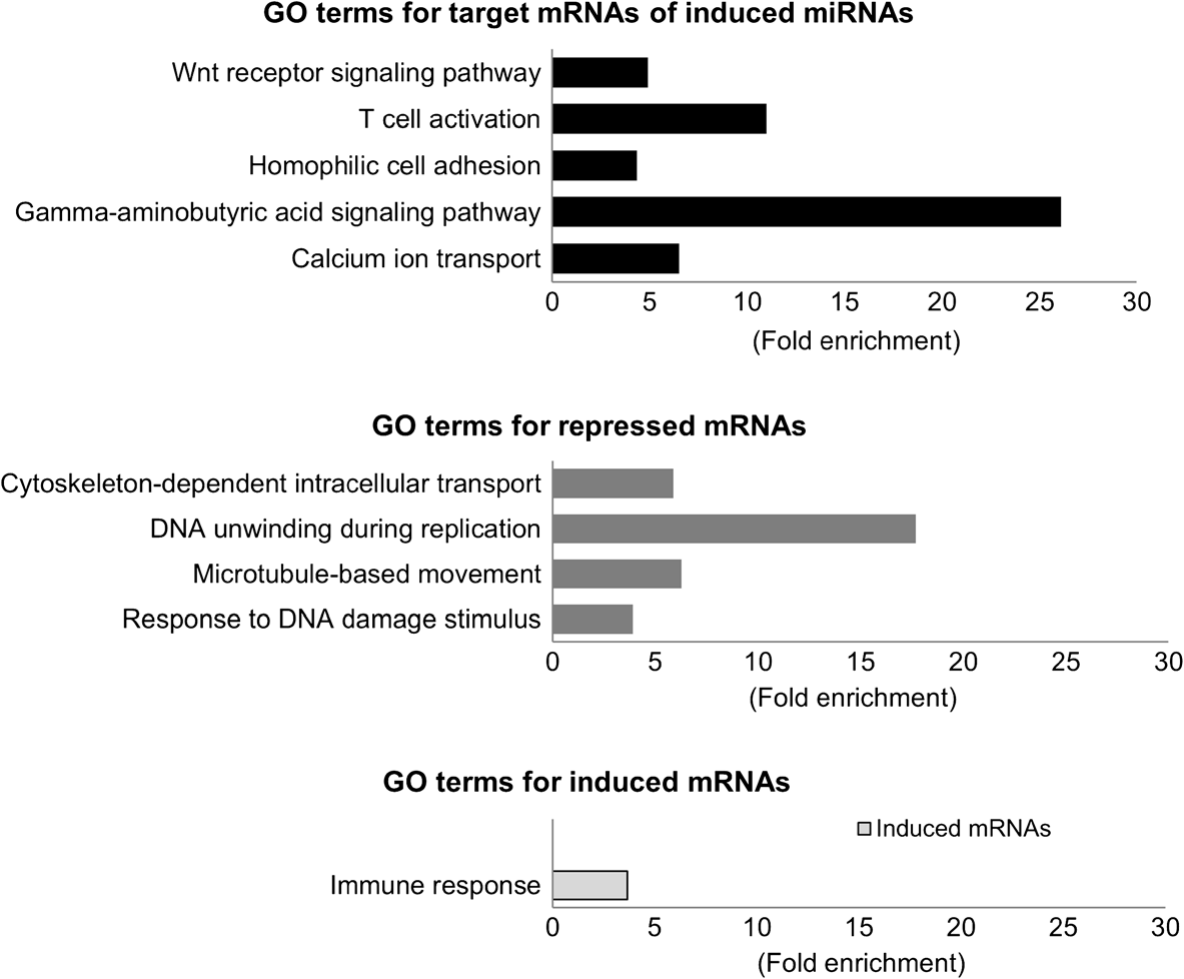
**Gene ontology (GO) annotation of differentially expressed genes and target genes of differentially expressed miRNAs between lungs of AIV infected and non-infected chicken in biological process category (P < 0.05).** Fold enrichment is a ratio obtained by dividing user’s percentage by the percentage of each category of the whole genome.

The significantly enriched functional terms in biological processes from differentially expressed host genes and predicted target genes of differentially expressed miRNAs with AIV infection, respectively, are presented in Additional file 3: Table S3. The immune related GO terms are presented in Figure 5. Developmental process (1.95 fold) was enriched by both target genes of repressed miRNAs and induced mRNAs. Two immune related GO terms, immune system process and immune response, were only enriched by induced host genes.

GO terms significantly enriched in repressed genes included four clusters: cytoskeleton-dependent intracellular transport, DNA unwinding during replication microtubule-based movement and response to DNA damage stimulus. For GO terms significantly enriched in targets of induced miRNAs, five GO terms were enriched. These included calcium ion transport; homophilic cell adhesion; T cell activation; gamma-aminobutyric acid signalling pathway; and Wnt receptor signalling pathway.

## Discussion

Integration of global profiling of miRNAs and mRNA expression may provide a unique opportunity in enhancing our understanding of regulatory mechanisms in many biological processes including virus infection. Identification of differentially expressed miRNAs associated with AIV infection is the first critical step of this comprehensive analysis. With limited number of biological replicates, and the nature of distribution of discrete measurement data other than continuous variable in microarray data, no single statistical method currently available can meet all conditions of this type of data [31]. As the main purpose of this step was to narrow down a small set of differentially expressed miRNAs that are potentially associated with AIV infection, our strategy was to include any potential important miRNAs (relative loose criteria with potential high false positive rate) for global enrichment analysis, but more conservative for further analysis for individual miRNA. Therefore, we chose three methods that have been intensively used for next generation sequencing data analysis [32-34], including Fisher’s exact test, DESeq and edgeR. Based on the results, Fisher’s exact test is less stringent than both DESeq and edgeR. On the other hand, both DESeq and edgeR might be too conservative. For example, gga-miR-206 was not identified by DESeq, neither by edgeR, but it was confirmed by real-time RT-PCR. This did suggest that the criteria used in the DESeq and edgeR analyses might miss some true positive, although this may exclude potential false positives. Thus, for the following global functional term enrichment analysis, our analysis was primarily based on the results from Fisher’s exact test. However, we are more interested in the 17 miRNAs (Tables 2, 3 and 4) that were identified as significant in at least two of the three methods of analysis, and especially the three miRNAs (gga-mir-1719, 1594 and 1599) identified across all three analysis. Of particular note, these three miRNAs are chicken specific miRNAs. To our knowledge, this is the first report of potential function study of these three miRNAs in animals. Further investigation of underlying mechanisms of these miRNAs in regulating AIV replication will expand our knowledge in host-pathogen interaction in animals.

Genetics play an important role in the regulation of miRNAs expression in animals [35]. We hypothesize that genetics affect the regulation of miRNA expression during AIV infection in the chicken. In the present study with broiler chickens, there were more miRNAs up-regulated than down-regulated with virus infection, which was the opposite with our previous miRNA profiling in layer chickens with AIV infection (more down-regulated than up-regulated) [20]. Table 8 lists differentially expressed miRNAs in both current broiler and previous layer studies. Only two (miR-1599 and miR-1416) of eighteen miRNAs had consistent directions of regulation following AIV infection, which suggests that these two miRNAs were conserved responses to AIV infection across diverse genetic lines. The major discrepancies between two studies might be due to: broilers and layers being genetically distinct chicken breeds with long-term diverse selection targeting on growth and egg production, respectively. Specifically, broilers are specialized in the production of a short term humoral response, while layers have a long-term humoral response in combination with a strong cellular mediated response [36]. The differential expression of miRNAs between them may reflect different miRNA-mediated host responses to virus infection. For example, differential expression of miR-142-3p in conventional CD4^+^ T cells and CD25^+^ T_REG_ cells in mice control the functions of both effector and suppressor cells. In the current study, gga-miR-142-3p was down-regulated in layers and up-regulated in broilers, which indicate that host immune response to AIV infection mediated by gga-miR-142-3p in broiler chickens may be different from layer. In summary, although other factors such as time of challenge may contribute to great miRNA difference between broilers and layers, these results strongly suggest that genetic backgrounds play a vital role in the regulation of miRNAs during AIV infection in chickens.

**Table 8:** Comparison between layer and broiler miRNA deep sequencing results (P < 0.05, Q < 0.05 and Ratio > 2)

	**miRNAs**	**Layers**			**Broilers**		
		**Infected**	**Non -infected**	**Ratio (Normalized)**	**Infected**	**Non -infected**	**Ratio (Normalized)**
Inconsistent	gga-mir-106	0	27	+	897	394	2.83
	gga-mir-142-3p	2	49	0.07	1055	461	2.84
	gga-mir-142-5p	2	49	0.07	1100	481	2.84
	gga-mir-144	111	94	0.21	13216	4727	3.47
	gga-mir-146a	7	105	0.12	1331	639	2.59
	gga-mir-15a	2	102	0.04	2431	861	3.48
	gga-mir-16-1	1	107	0.02	2724	1206	2.80
	gga-mir-1729	0	24	+	4292	843	6.32
	gga-mir-19b	1	31	0.06	955	181	6.55
	gga-mir-193a	5	26	0.35	1230	461	3.31
	gga-mir-206	101	9	20.38	28	98	0.35
	gga-mir-20a	1	23	0.08	426	190	2.78
	gga-mir-20b	0	10	+	734	378	2.41
	gga-mir-223	1	15	0.12	1842	717	3.19
	gga-mir-29a	2	15	0.24	205	108	2.36
	gga-mir-451	93	1287	0.13	207487	35518	7.25
Consistent	gga-mir-1599	48	13	6.17	184	7	32.64
	gga-mir-1416	17	10	3.09	25	12	2.59

Note: ^+^ Specifically expressed in non-infected lungs.

The miR-155 has been reported to play important roles in both innate and adaptive immunity in mammals [7,37,38]. miR-155 knock-out mice are not capable of generating defensive immune responses, developing lymphocytes, or antigen-presenting cell functions [39]. The up-regulation of miR-155 with poly (I:C) and IFNβ stimulation in mouse macrophages suggest an important role of miR-155 in the regulation of viral infection [37]. In the current study, gga-miR-155 was significantly induced by AIV infection, which was consistent with other studies [40]. Based on target prediction, miR-155 could target the chicken anti-influenza gene MX1, therefore playing a role in host and AIV interactions in chickens. The activation of c-Jun NH_2_-terminal kinases (JNK) pathway can eliminate virus-infected cells by apoptosis. The inhibition of JNK pathway blocked the expression of miR-155 in murine macrophages [37,40]. Down-regulation of TNFRSF19 (TNF receptor superfamily member 19), one of key genes in JNK pathway, indicate that antiviral activities through JNK pathway might be inhibited. Therefore, up-regulated miR-155 might also activate JNK pathway, and subsequently induce apoptosis to eliminate virus infected cells [37,40].

Most of the up-regulated miRNAs were predicted to target the hemaglutinin (HA) and neuraminidase (NA) mRNAs such as miR-34a and miR-155. Both HA and NA are major surface glycoproteins. HA is responsible for receptor binding and virus fusion [41], while NA is responsible for receptor destruction and virion release [41]. Therefore, induction of these miRNAs might affect virus attachment and release and therefore the formation of new infectious viral particles. In addition, three down-regulated miRNAs (miR-206, miR-301 and miR-187) also were predicated to target on AIV genome. The first line of evidence from this integrative analysis strongly indicates that the importance of several candidate miRNAs including miR-34a, 146a, 155 and 206 warrant further investigation to understand the mechanisms of miRNA regulation of AIV infection in chickens.

## Conclusions

In summary, this comprehensive analysis has provided several lines of new evidence on how host miRNA might regulate host response to AIV replication in broilers. Specifically, this study generated a list of strong candidate miRNAs including miR-34a, 122–1, 122–2, 146a, 155, 206, 1719, 1594, 1599 and 460 that potentially regulate AIV infection in chickens. In addition, several candidate genes including MX1, IL-8, IRF-7, TNFRS19 have been identified to be associated with AIV infection in broilers. Finally, comparison with our previous layer miRNA profiling, this study strongly indicates that genetic background is a critical factor in determining miRNA abundance and regulation during AIV infection. As main focus of this global profiling analysis was to generate new hypothesis by screening whole genome miRNAs and mRNAs, our on-going effort using experimental approach such as knock-down or over-express candidate miRNAs and mRNAs *in vitro* is expected to provide new evidence in understanding these regulatory mechanisms of AIV infection in chickens.

## Methods

### Sample collection and RNA isolation

Day old broilers (Cobb-Vantress, Inc.) were randomly divided into two groups (4 chickens per group), housed in negative pressure Horsfall-Bauer, temperature control isolation units and provided with water and commercial feed *ad libitum*. At one week of age, one group was inoculated with 0.1 ml of CK/TX/02/H5N3 virus containing10^7.5^ EID_50_/ml and the remaining chickens were inoculated with PBS (mock treatment) by the intra-choanal cleft route. At 4 days post-inoculation (dpi), depression and severely congested lungs were observed in the treated chickens. Therefore, all chickens were humanely euthanized at 4 dpi, and lungs were collected for RNA isolation. The animal experiment was performed according to the guidelines approved by the Institutional Animal Care and Use Committee, Texas A&M University.

Two pools of total RNA samples (2 random chickens per pool) were generated from the infected and non-infected group. Total RNAs were isolated using Trizol (Invitrogen, Carlsbad, CA) following the manufacturer’s protocol. Dnase I (Ambion, Austin, TX) digestion was carried out after RNA isolation according to manufacturer’s instructions. RNA concentration and purity were determined by measuring absorbance at 260 nm and A260/A280 ratio using a NanoDrop ND-1000 spectrophotometer (Nanodrop Technologies, Wilmington, DE). RNA samples were stored at −80°C until further use.

### Viral titration

Virus titers in lungs of inoculated chickens were determined at 4 dpi by real-time RT-PCR of influenza virus matrix gene using AgPath-ID™ AIV- M kit (Ambion, Austin, TX) following the manufacturer’s instructions. For quantitation of virus load, RNA was extracted from serially diluted H5N3 virus stock (10^1.5^–10^5.5^ log_10_ EID_50_/ml) and used to generate a standard curve. The amount of RNA in the samples was converted into log_10_ EID_50_/ml by interpolation as described previously [42].

### Small RNA sequencing and analysis

For small RNA library construction, total RNA samples from lungs of infected and non-infected broiler chickens were prepared using the DGE-Small RNA Sample Prep Kit (Illumina, San Diego, CA) as previously described [20]. A total of two Solexa-ready small RNA templates were analyzed on an Illumina 1 G Genome Analyzer at the University of Houston. Cluster generation was performed and clusters were sequenced. Initial sequence process and analysis was done as previously described [20]. All unique sequence reads with a minimum read count of 5 were aligned with precursor chicken miRNA sequences from miRBase version 16 [22-24]. Reads of each miRNA were the sum of exact and loose matches (± 4 bp) to known miRNAs. For each sample, counts were normalized to the total number of small RNA sequences, and then for each miRNA, the normalized number of counts was compared between groups. False discovery rate (FDR) (Q values) was calculated by R program according to Benjamin’s method [43]. Fisher’s exact test, DESeq [33] and edgeR [32] were used to identify differentially expressed miRNAs (*P* < 5%). Fold changes for Fisher’s exact test were calculated as the ratio of normalized reads of infected over non-infected group. Statistics related to over representation of functional categories were performed using DAVID [44-46]. A P < 0.05 was considered significant. Novel miRNAs were identified using the methods of Creighton et al. and Wang et al. [20,47].

#### Confirmation of miRNA expression by northern-blot

Expression of one potential novel miRNA was confirmed by Northern blot analysis using the same total RNA samples as those used for small RNA library construction. Total RNA of infected and non-infected lung samples (15 μg each) were separated on a 15% denaturing acrylamide gel and transferred onto a GeneScreen Plus nylon membrane (GE Healthcare, Piscataway, NJ). Membranes were fixed by UV cross-linking at 1200 μJ and baking at 80°C for 1 hour. DNA probes (antisense to two mature miRNA sequences) were end-labeled with [γ-^32^P] ATP (GE Healthcare, Piscataway, NJ) using a mirVana Probe & Marker Kit (Ambion, Austin, TX). Pre-hybridization, hybridization and washes were carried out at 42°C using ULTRAhyb-Oligo hybridization buffer according to the manufacturer’s instructions (Ambion, Austin, TX). Chicken U6 small nuclear RNA was used as an internal control to account for loading differences between samples.

#### Confirmation of differentially expressed miRNAs by TaqMan miRNA assay

To determine the expression of miRNAs by quantitative RT-PCR (qRT-PCR), TaqMan miRNA assays were performed. The specific stem-loop RT primers for miR-206, miR-451 and U6 were obtained commercially from Applied Biosystems (Foster City, CA). In brief, cDNA was synthesized from total RNA by using the miRNA specific primers according to the protocol of TaqMan Micro RNA Assays (Applied Biosystems, CA). Reverse transcriptase reactions contained 10 ng of RNA samples, 3 μl of 50 nM stem loop RT primer and reagents from the TaqMan MiRNA Reverse Transcription Kit (Applied Biosystems, CA). The 15 μl reactions were incubated for 30 min at 16°C, 30 min at 42°C and 5 min at 85°C, and then held at 4°C. Real-time PCR was performed using gene specific probes and a pair of primers and TaqMan 2X Universal PCR Master Mix (No AmpErase UNG) (Applied Biosystems, CA). The 20 μl PCR reactions included 1.33 μl cDNA products, 10 μl PCR master mix, and 1 μl 20X TaqMan MiRNA Assay mix (Applied Biosystems, CA). These reactions were incubated at 95°C for 10 min, followed by 40 cycles at 95°C for 10 s, 60°C for 40 s and 72°C for 1 s using an ABI 7900 Realtime PCR instrument (Applied Biosystems, CA). All reactions were run in triplicate. The threshold cycle was defined as the fractional cycle number at which the fluorescence passes the fixed threshold. The expression levels of miR-206 and miR-451 in each sample were measured in terms of threshold cycle value and normalized to U6 expression using 2^-∆∆CT^[48].

### miRNA prediction and validation

#### Target prediction

The chicken (*Gallus gallus*) Unigene database (NCBI) and the miRNA target prediction algorithm miRanda 3.1 ( http://www.microran.org/microrna/getDownloads.do) were employed to predict potential targets of all the differentially expressed miRNAs. For miRanda, default parameters were used with the following exceptions: the score was set to ≥ 130 and the free energy was set to ≤ −16 kCal/mol. The predicted targets were further filtered using more stringent criteria in which they must contain either (1) a match between nucleotides 2–8 of the miRNA with the target sequence or (2) a match between nucleotides 2–7 and 13–16 of the miRNA with the target sequence (G:U base-pairing was tolerated). A set of target genes containing miR-146a binding sites within their 3Â´UTRs were selected for further analysis using a dual luciferase reporter assay.

#### Insertion of target sequences into psiCHECK-2

For each potential target gene, the region of 3Â´UTR flanking the miR-146a binding sites was PCR amplified from Red Jungle Fowl genomic DNA using gene specific primers (Additional file 4: Table S4). Each PCR product was cloned into the 3Â´UTR of the *Renilla* reporter gene in the psiCHECK-2 vector (Promega, WI) using NotI and XhoI restriction sites from the multicloning site.

#### Construction of RCAS viruses expressing chicken miR-146a

The previous described RCASBP(A)-miR vector [49] was used to ectopically express miR-146a. In order to produce RCAS viruses expressing chicken miR-146a, an entry vector was constructed using PAGE purified 76-nt forward and 68-nt reverse oligos (Invitrogen). Restriction sites for SphI and NgoMIV were introduced at the 5Â´- and 3Â´-ends, respectively. Forward and reverse oligos were mixed at a final concentration of 1 μM, denatured at 95°C for 20 sec and annealed at RT to generate a short double-stranded DNA fragment. The fragment was then cloned into the pENTR3C-miR-SphNgo vector at the SphI and NgoMIV restriction sites. The RCASBP(A)- miR-146a vector was generated via a recombination between the pENTR3C- miR-146a entry vector and RCASBP(A)-YDV gateway destination vector using a LR clonase kit (Invitrogen, CA). To produce miR-146a expressing viruses (RCAS- miR-146a), the RCASBP(A)-miR146a plasmid vector was transfected into DF-1 cells, a chicken embryo fibroblast continuous cell line, using FuGENE 6 (Promega, WI). Virus stock was harvested at day 6 post transfection and titer was determined using immunofluorescence staining with the monoclonal 3 C2 antibody against the RSV/ALV gag protein (Developmental Studies Hybridoma Bank, University of Iowa) and FITC-conjugated goat anti mouse IgG (Invitrogen, CA). In addition, RCAS viruses (RCAS-*SC*) expressing a scrambled control sequences were produced to serve as a negative control. Ectopic expression of the miR-146a was validated using a miScript Reverse Transcription kit and a miScript SYBR Green PCR kit (Qiagen, CA).

#### Dual luciferase reporter assay

DF1 cells were infected with either RCAS-miR-146a or RCAS-*SC* at a multiplicity of infection of 1 and maintained for 6 days in a 96-well plate in RPMI 1640 medium supplemented with 1% heat-inactivated FBS, L-glutamine, penicillin (100 U/ml), streptomycin (100 μg/ml), and fungizone (4 μg/ml), at 37°C with 5% CO_2_. The psiCHECK-2 construct (100 ng) for each potential target gene, as well as the scramble control, were then transfected into both RCAS-*miR-146a* or RCAS-*SC* infected DF-1 cells using FuGENE 6 (Promega, WI). Forty-eight hours post-transfection, cells were washed with PBS and lysed in Passive Lysis Buffer (Promega, WI). For each transfection, firefly and Renilla luciferase activities were determined using the Dual-Luciferase Reporter Assay System (Promega, WI) and a VictorLight 1420 luminescence counter (PerkinElmer, MA). The Renilla luciferase signal was normalized to the firefly luciferase signal. The normalized Renilla luciferase activity was compared between the RCAS-miR-146a and the RCAS-SC using student’s *t*-test (P < 0.05). Triplicates for each target construct were performed and the assay was repeated to confirm the results.

### Microarray analysis

Microarray experiment design: Four biological replicates from infected and non-infected groups were used with dye balance in order to prevent dye-bias during sample labeling.

Labeling and hybridization: The integrity of total RNA samples was confirmed using Agilent Bioanalyzer 2100 Lab-on-chip system (Agilent Technologies, Palo Alto, CA). Four hundred nano-grams (ng) of total RNA were reverse-transcribed to cDNA during which a T7 promoter sequence was introduced into the cDNA. T7 RNA polymerase-driven RNA synthesis was used for preparation and labeling of RNA with Cy3 (or Cy5) dye. Fluorescent cRNA probes were purified using Qiagen RNeasy Mini Kit (Qiagen, Valencia, CA), and an equal amount (825 ng) of Cy3 and Cy5 labeled cRNA probes were hybridized to a 44 K chicken Agilent array (GEO accession: GSE9416). The hybridized slides were washed using a commercial kit package (Agilent Technol-ogies, CA) and then scanned using a Genepix 4100A scanner (Molecular Devices Corporation, Sunnyvale, CA) with a tolerance of saturation setting of 0.005%.

Microarray data collection and analysis: For each channel, the median of the signal intensity and local background values were used. A Locally Weighted Linear Regression (LOWESS) normalization was applied to remove signal intensity-dependent dye bias for each array using R program. A mixed model that included the fixed effects of dye (cy3 and cy5) and random effect of slide and array was used to analyze the normalized data by SAS (SAS institute, Cary, NC). P < 0.05 was considered significant. These data have been deposited in GEO (Accession numbers: GSM879919, GSM879925, GSM879936, GSM879937).

### Gene ontology

Functional annotations for differentially expressed genes were performed through the use of the Database for Annotation, Visualization and Integrated Discovery (DAVID) [44-46]. Statistics related to over representation of functional categories was based upon a Fisher’s Exact statistic methodology similar to that described by Al-Shahrour et al. [50]. A P < 0.05 was considered as significant.

## Competing interests

The authors declare that there are no competing interests.

## Authors' contributions

YW carried out the RNA isolation, small RNA library construction preparation, microarray analysis, analyzed data and drafted the manuscript. VB was responsible for the animal trial. BL and SR contribute to experiment design. BY, AB and PG contributed to the running of miRNA deep sequencing and analysis of miRNA. HL and NT developed the miRNA target confirmation; NI contributed to miRNA northern-blotting; RO provided experimental animals; HZ provided the concepts of the study, designed the experiment and revised the manuscript. All authors submitted comments, read and approved the final manuscript.

## Supplementary Material

Additional file 1 **Table S1.** The Novel miRNA. Click here for file

Additional file 2 **Table S2.** Immune related targets of differentially expressed miRNAs. Click here for file

Additional file 3 **Table S3.** Enriched GO terms in biological process of each comparison. Click here for file

Additional file 4 **Table S4.** Primer table. Click here for file
