# Supplementary material for: Integrated analysis of microRNA expression and mRNA transcriptome in lungs of avian influenza virus infected broilers
Source: BMC Genomics. 2012 Jun 22;13:278. doi: 10.1186/1471-2164-13-278 (PMC3496578; doi:10.1186/1471-2164-13-278)
Supplement: Additional file 4 — Table S4. Primer table. [file 1471-2164-13-278-S4.docx]

Primers used for miR-146a target validation

| Symbol/  GI | Primer (5´-3´) | Product length (bp) |
| --- | --- | --- |
| HIF1AN/  118092762 | F: GCACTCGAGAGGTACAAAGCCTCCTTTA  R: AAAGCGGCCGCACTCCAAATCAGGAACAGC | 459 |
| PDHB/  118097022 | F: GCACTCGAGCTGAAAGTTTTGAATGGAGC  R: AAAGCGGCCGCTACAGCAGAGTGGTCCTTT | 500 |
| LATS1/  118088356 | F: GCACTCGAGAATGGCAGTTCTTCTTTTGC  R: AAAGCGGCCGCGCTACAGCATTACTGATGTCAA | 315 |
| POU1F1/  45383513 | F: GCACTCGAGCACTTTACATTCCCACTCTCT  R: AAAGCGGCCGCTGCACACACAGCTGATAGA | 731 |
| CHMP2B/  71896762 | F: GCACTCGAGTCTGTTTCCGTGAAGGTTTT  R: AAAGCGGCCGCGTGCTTTTGCTGTTTCTGC | 250 |
| ARL11/  118084874 | F: GCACTCGAGGCTACAGAGATGGTAGGAGA  R: AAAGCGGCCGCCGTGCTTGAGCACTATTTTA | 271 |
| MAP3K3/  118102843 | F: GCACTCGAGAACTCAGCTGTCCTAGTCCT  R: AAAGCGGCCGCAGTGTAGCTCTTCCAGTTCC | 264 |
| miR-146a  (RT-PCR) | F: TGAGAACTGAATTCCATGGGTT  R: miScript Universal Primer (Qiagen) | - |
| miR-146aSC  (RT-PCR) | F: GCATATTAGTAGTGCGAACTGT  R: miScript Universal Primer (Qiagen) | - |
| miR-146a  (RCAS) | F: CGCACCCATGGAATTCAGTTCTCATAGTGAAGCAGCAGATGGTATGAGAACTGAATTCCATGGGTTTG  R: CCGGCAAACCCATGGAATTCAGTTCTCATACCATCTGCTGCTTCACTATGAGAACTGAATTCCATGGGTGCGCATG | - |
| miR-146aSC  (RCAS) | F: CGCCAGTTCGCACTACTAATATGCTAGTGAAGCAGCAGATGGTAGCATATTAGTAGTGCGAACTGTTG  R: CCGGCAACAGTTCGCACTACTAATATGCTACCATCTGCTGCTTCACTAGCATATTAGTAGTGCGAACTGGCGCATG | - |
